# Quantification of Anti‐Ro52, Anti‐Ro60 and Anti‐La Autoantibodies at High Sample Dilution and Their Predictive Value for Congenital Heart Block

**DOI:** 10.1111/sji.70164

**Published:** 2026-11-03

**Authors:** Lisa Rudolph, Elina Richardsdotter Möller, Rui Da Silva Rodrigues, Gregoire Martin de Fremont, Vijole Ottosson, Linda Lagnefeld, Iva Gunnarsson, Elisabet Svenungsson, Felicia Nordenstam, Gunnar Bergman, Sven‐Erik Sonesson, Gudny Ella Thorlacius, Marie Wahren‐Herlenius

**Affiliations:** ^1^ Division of Rheumatology, Department of Medicine Karolinska Institutet, and Karolinska University Hospital Stockholm Sweden; ^2^ Department of Clinical Immunology and Transfusion Medicine Karolinska University Hospital Stockholm Sweden; ^3^ Pediatric Cardiology Unit, Department of Women's and Children's Health Karolinska Institutet, Karolinska University Hospital Stockholm Sweden; ^4^ Broegelmann Research Laboratory, Department of Clinical Science University of Bergen Bergen Norway

**Keywords:** autoantibodies, congenital heart block, La, Ro52, Ro60

## Abstract

Maternal anti‐Ro/La antibodies confer a risk of congenital heart block (CHB), but current methods for predicting risk relying on the presence or absence of these autoantibodies perform poorly. To identify more accurate biomarkers of CHB risk, we investigated the association between maternal levels of autoantibodies and the risk of CHB. A total of 318 women positive for anti‐Ro/SSA autoantibodies were included. Autoantibody presence was obtained from health records for all, and ELISA was used to quantify levels of anti‐Ro52, anti‐Ro60, and anti‐La in a subset of 116 women with samples obtained during pregnancy, including 27 CHB‐affected pregnancies. Predictive models for CHB risk were developed using logistic regression and machine learning. Anti‐Ro/SSA positive women with anti‐Ro60 were more likely to have pregnancies with CHB (*p* < 0.05). Levels of both anti‐Ro60 (*p* < 0.001) and anti‐Ro52 (*p* < 0.05) were significantly higher in CHB‐affected pregnancies and correlated (*r* = 0.8, *p* < 0.0001), while levels of anti‐La did not differ between groups. Levels of anti‐Ro60 were identified as the strongest individual predictor of CHB, with a relative risk of 16.3 (95% CI 1.04–255.0, *p* < 0.001) for the top versus the bottom decile. In all tested combinations, models that included anti‐Ro60 levels outperformed models that did not. Models based on anti‐Ro60 levels alone reached an area under the curve (AUC) of 0.76 and an odds ratio (OR) per standard deviation increase of 2.8 (95% CI 1.6–5.3). These findings confirm a strong association between maternal anti‐Ro antibody levels and CHB risk in anti‐SSA‐positive pregnancy, highlighting anti‐Ro60 autoantibody levels as a valuable biomarker in risk stratification.

## Graphical abstract

Autoantibody levels identify pregnancies at risk of congenital heart block.

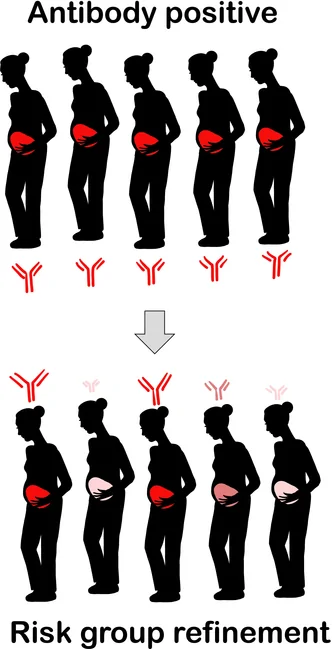

## 1 Introduction

Autoimmune congenital heart block (CHB) is the most severe manifestation of neonatal lupus erythematosus (NLE), a condition mediated by transplacental transfer of maternal autoantibodies targeting the Ro52, Ro60, and La proteins. CHB typically presents between gestational Weeks 18–24 as a fetal atrioventricular (AV) block in the absence of structural cardiac abnormalities. In some cases, cardiac involvement can also manifest as endocardial fibroelastosis, whereas myocarditis or cardiomyopathy may develop in later stages.

Maternal autoantibodies against Ro52 and/or Ro60 (collectively referred to as anti‐SSA) and La (anti‐SSB) are detectable in 0.5%–1% of the general population and are commonly associated with autoimmune conditions such as systemic lupus erythematosus (SLE) and Sjögren's disease [1, 2, 3, 4]. These diseases disproportionately affect women and are often diagnosed during or after reproductive age. Although the majority of pregnancies in anti‐Ro/SSA positive women are uncomplicated, CHB develops in approximately 2%–3% of the pregnancies [5, 6]. The recurrence risk in a subsequent pregnancy was reported at 12.1% and 12.5% in the two available studies performed in a population‐based manner [6, 7], while a review of studies concluded by 2015 suggested a mean recurrence rate of 15.8% [5]. The exact recurrence rate may differ between study populations but stays far below 100%, suggesting a multifactorial pathogenesis involving both maternal and fetal risk factors.

Historically, CHB has been associated with a high mortality rate of 20%–30% [8, 9]. However, recent evidence shows that early detection and intervention can significantly improve outcomes. Specifically, enrolling anti‐Ro/SSA positive women in surveillance programs, including weekly fetal Doppler echocardiography during gestational weeks 18–24, and initiating fluorinated steroid therapy upon early signs of AV block (Grade II or III) or endocardial fibroelastosis reduced mortality to 3.8% [6]. These findings underscore the importance of timely diagnosis and treatment. Given the low overall risk of CHB at 2%–3% in anti‐Ro/SSA positive pregnancies [5, 10], the routine use of intensive surveillance poses a psychological burden for expectant families and incurs substantial healthcare costs [11]. There is, therefore, a pressing need for more accurate biomarkers to identify pregnancies at high risk for CHB and to guide clinical decision‐making. Several studies have suggested that levels of the Ro/SSA and La/SSB autoantibodies may relate to the risk of CHB [12, 13, 14], and to address the need for clinically useful biomarkers, we here investigated whether maternal levels of anti‐Ro52, anti‐Ro60, and anti‐La autoantibodies, and their combinations, are associated with CHB in a national and a prospectively collected cohort. Our goal was to evaluate their utility as predictive biomarkers for CHB risk stratification in clinical practice.

## 2 Methods

### 2.1 Study Population

Women positive for anti‐Ro/SSA and/or La/SSB autoantibodies during pregnancies with or without CHB were included from two sources: a previously described national CHB registry cohort [15], and a prospectively followed cohort of anti‐Ro/SSA‐positive pregnant women attending the Paediatric Heart Centre Stockholm‐Uppsala, Karolinska University Hospital/Uppsala University Hospital, Sweden, 2000–2020. For the latter cohort, anti‐Ro52 antibodies served as an inclusion criterion for the major part of the period. In total, 318 women with available clinical data on anti‐Ro52, anti‐Ro60, and anti‐La autoantibody positivity from routine testing were included in the present study.

Based on whether the women had ever experienced a CHB pregnancy characterized by AV block Grade II–III and/or endocardial fibroelastosis confirmed by a fetal cardiologist, women were assigned into either the CHB_ever_ or CHB_never_ groups (Table 1). At least one sample from an ongoing pregnancy was available for 116 women, including 27 with CHB‐affected pregnancies. In 22 of these CHB‐affected pregnancies, samples had been taken before administration of fetal steroids. Longitudinal samples spanning one or more pregnancies from six women were included. A schematic flow chart detailing the samples included is shown in Figure 1. In the three multiple pregnancies included, only one fetus per pregnancy was affected by CHB. Clinical characteristics of the CHB_ever_ subgroup that had samples collected during pregnancy and before steroid treatment are shown in Table 2.

**TABLE 1:** Clinical characteristics of the total number of anti‐Ro/La antibody‐positive women and of anti‐Ro/La antibody‐positive women with samples during pregnancy.

	CHB_never_	CHB_ever_
Individuals, *n*	199	119
Individuals with pregnancy samples and no FS, *n*	89	22
Maternal age in years at first sampled pregnancy, range (mean)	22–47 (33.0)	23–41 (31.7)
Maternal primary autoimmune diagnosis at time of first		
Sampled pregnancy, *n*		
Sjögren's disease	43	4
Systemic lupus erythematosus	30	3
Other	13	2
Multiple pregnancy		
Twins	0	2
Triplets	0	1
Gestational age at index pregnancy sampling		
GW < 18	13	0
GW 18–24	66	16
GW ≥ 25	6	6

Abbreviations: CHB, congenital heart block; FS, fetal steroids; GW, gestational week.

**FIGURE 1:**
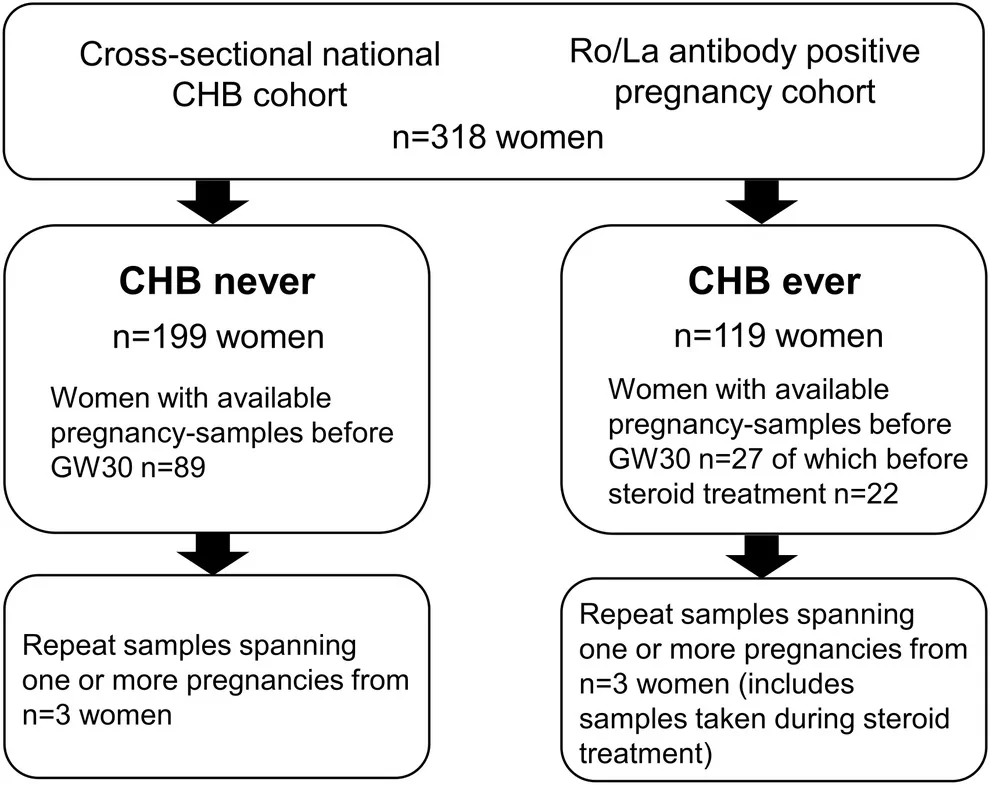
Schematic flow chart of cohorts and samples included.

**TABLE 2:** Features of the CHB_ever_ group with pregnancy samples before fetal steroid treatment.

Individuals, *n*	22
Gestational age at CHB diagnosis	
GW 18–24	15
GW ≥ 25	7
Fetal diagnosis^a^, *n*	
AV‐block II	3
AV‐block III	18
Endocardial fibroelastosis	2
Recurrent CHB pregnancy, *n*	
Yes	2
No	20

Abbreviations: AV, atrioventricular; CHB, congenital heart block; GW, Gestational week.

^a^ Highest degree of AV block observed.

The study was approved by the Regional Ethics Committee Stockholm, and written informed consent was obtained from all participants.

### 2.2 Autoantibody and IgG Analyses

Presence of anti‐Ro52, anti‐Ro60, and anti‐La autoantibodies in serum was analysed in clinical routine with the BioPlex 2200 multiplex immunoassay (Bio‐Rad Laboratories, Hercules, CA, USA) at the Division of Clinical Immunology, Karolinska University Hospital, Sweden.

Levels of anti‐Ro52, anti‐Ro60, and anti‐La autoantibodies in plasma were determined by ELISA (ORG 652, ORG 660, and ORG 509, Orgentec). In short, samples diluted in 0.05% Tween‐20 in PBS (TPBS) with 1% milk powder were added to the precoated plates and incubated for 2 h. A polyclonal ALP‐conjugated rabbit anti‐human IgG antibody (Dako) diluted 1:2400 in TPBS with 1% milk powder was used for detection of bound autoantibodies and incubated for 1 h. Phosphatase substrate tablets (S0942, Sigma) dissolved in substrate buffer pH 9.6 were added to plates and incubated in the dark for 120 min before measuring absorbance at 405 nm with a spectrophotometer (Molecular Devices). The volume added to each well was 100 ul; samples were tested in duplicate, and all incubations were performed at room temperature. Washing with wash buffer (0.05% Tween‐20, NaCl 9 g/L in dH_2_0) was performed three times between each step. For one participant, serum was used in place of plasma.

Total IgG Concentrations were Measured Using a Separate ELISA (Human IgG ELISAbasic, Mabtech) in a Similar Manner, with Plasma Diluted 1:100,000 and Absorbance Measured after 60 min of Substrate Incubation.

### 2.3 Statistical Analysis and Predictive Modelling

Categorical variables were compared using the Chi‐square test, while continuous variables compared between two independent groups were analysed using the Mann–Whitney U‐test and Spearman rank correlation. To identify autoantibody profiles associated with case status (CHB_ever_ vs. CHB_never_), and to assess their predictive performance, we applied logistic and least absolute shrinkage and selection operator (LASSO) machine learning regression models. Anti‐Ro52, anti‐Ro60, and anti‐La autoantibody levels were evaluated individually and in all possible combinations, with and without inclusion of total IgG levels. Model performance was assessed using repeated stratified 5‐fold cross‐validation (50 repeats), with discrimination quantified by the area under the receiver operating characteristic curve (AUC). We selected the simplest model for which the cross‐validated AUC was within one standard error of the best‐performing model. ROC curves and 95% confidence intervals (DeLong method) were calculated from model predictions. Because of the limited number of cases and potential separation, Firth's penalized logistic regression was used as a sensitivity analysis to obtain bias‐reduced estimates of relative risk and confidence intervals. Risk stratification was visualized by grouping predicted probabilities into deciles and comparing observed case proportions across risk strata. Association strength was assessed using logistic regression. For each antibody combination, a standardized composite model score was derived from log‐transformed and standardized antibody measurements. Odds ratios (ORs) with 95% confidence intervals were calculated for a one standard deviation increase in model score.

All statistical analyses were performed in GraphPad Prism version 10.6.1 or R using the pROC, glmnet, and binom packages. A *p* < 0.05 was considered statistically significant. Bonferroni correction was applied for multiple comparisons when applicable.

## 3 Results

### 3.1 Anti‐Ro60 Antibodies Are More Common in Anti‐Ro/La Positive Women Who Experienced at Least One CHB Pregnancy

Anti‐Ro/La autoantibodies define pregnancies with a risk of CHB, but only 2%–3% of the pregnancies are affected [5, 10]. To investigate if any specificity or level thereof could serve as a biomarker of CHB risk in the Ro/La autoantibody‐positive pregnancies, we first compared the presence of autoantibody specificities as determined by clinical routine assays between mothers who had never experienced a CHB pregnancy (CHB_never_) and those who had (CHB_ever_). The analysis revealed that the anti‐Ro60 specificity was more common in anti‐Ro/La positive women who had experienced a CHB pregnancy compared to those who had not (95.0% vs. 87.4%, *p* < 0.05) (Figure 2A).

**FIGURE 2:**
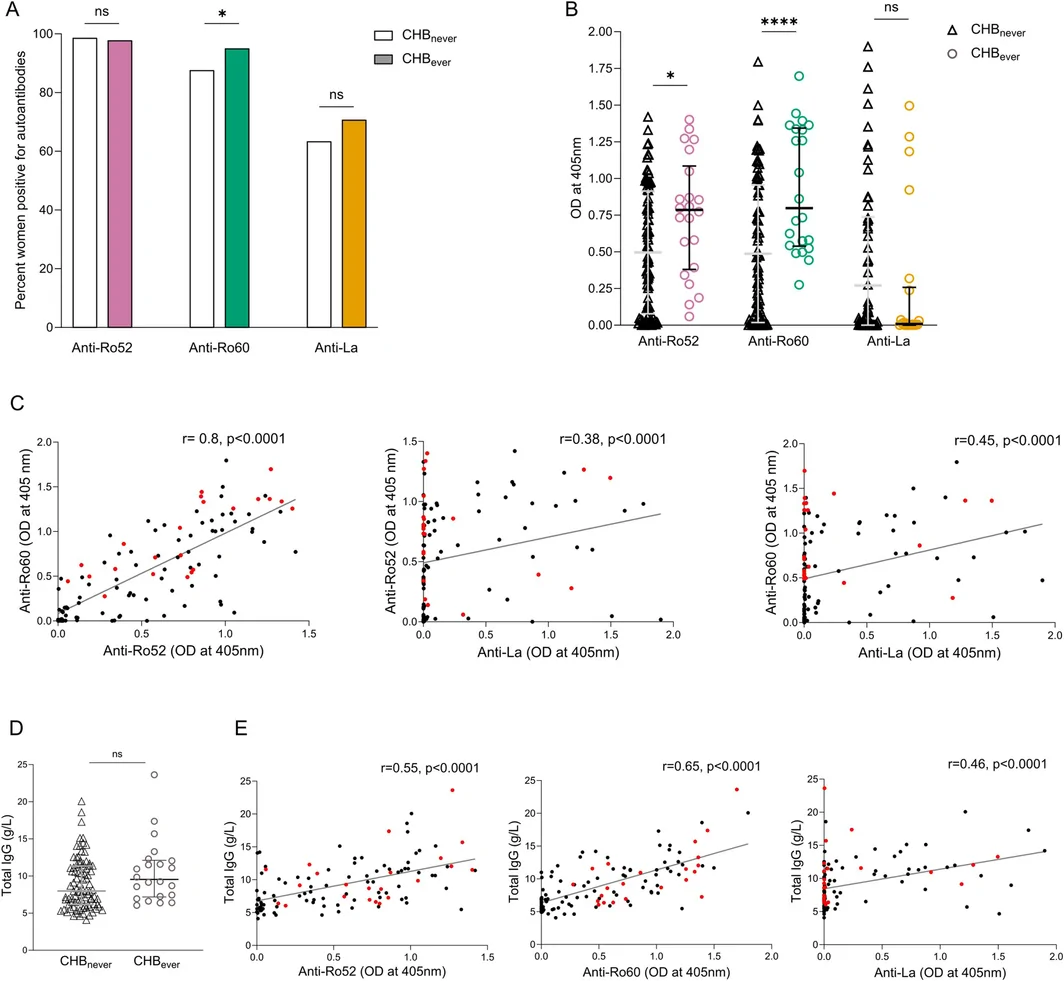
Distinct patterns of autoantibodies and total IgG levels. (A) Positivity for anti‐Ro52, anti‐Ro60, and anti‐La autoantibodies in CHB_ever_ and CHB_never_ groups based on clinical routine testing (*n* = 318). *p < 0.05 (B) Autoantibody levels (optical density, OD) for anti‐Ro52, anti‐Ro60, and anti‐La in samples obtained during pregnancy (earliest sample closest to GW 18–24 available), measured by ELISA at 1:10,000 (anti‐Ro60) or 1:2000 (anti‐Ro52 and anti‐La) dilution. Individuals in the CHB_ever_ group had not been treated with fluorinated steroids when the sample was obtained. *p < 0.05, ****p < 0.0001. (C) Spearman rank correlation coefficient (r) between levels of anti‐Ro52, anti‐Ro60, and anti‐La during pregnancy (earliest sample closest to GW 18–24 available) (*n* = 111). CHB_ever_ samples indicated in red. (D) Total IgG levels during pregnancy (earliest sample closest to GW 18–24 available) measured by ELISA in CHB_ever_ and CHB_never_ groups. (E) Spearman rank correlation coefficient (r) between total IgG levels and levels of anti‐Ro52, anti‐Ro60, and anti‐La, respectively. CHB_ever_ samples indicated in red.

### 3.2 Anti‐Ro60 and Anti‐Ro52 Autoantibody Levels Are Higher in CHB_ever_ Mothers Than in CHB_never_ Mothers

We next examined whether the levels of anti‐Ro52, anti‐Ro60, and anti‐La autoantibodies differed between the CHB_never_ and CHB_ever_ groups. Pregnancy may affect IgG levels, and we therefore restricted the analysis to samples obtained before week 30 of pregnancy and included only samples taken before administration of fetal steroid treatment. Samples from 89 women of the CHB_never_ and samples from 22 women of the CHB_ever_ group were available for such analysis. For the experiments, a range‐finding test for sample dilution was first performed to ensure measurements in high titre samples could be performed within the linear part of the curve. These titrations established dilutions for testing of anti‐Ro60 at 1:10,000, while anti‐Ro52 and anti‐La only required sample dilutions at 1:2000, indicating that anti‐Ro60 antibodies appear at much higher titres than anti‐Ro52 and anti‐La autoantibodies.

Compared to CHB_never_ pregnancies, samples from CHB_ever_ pregnancies had significantly higher levels of anti‐Ro60 and anti‐Ro52, whereas anti‐La levels did not differ between the groups (Figure 2B). Among the three autoantibodies, anti‐Ro60 levels had the strongest statistical association with CHB status (Figure 2B).

Correlation analyses revealed a strong positive correlation between anti‐Ro52 and anti‐Ro60 levels (*r* = 0.8, *p* < 0.0001), whereas their correlation with anti‐La was at most moderate with *r*‐values at 0.4–0.5 (Figure 2C).

Given that all three autoantibodies were detected as IgG isotype immunoglobulins, we assessed whether total IgG levels differ between CHB_ever_ and CHB_never_ pregnancies to understand if high IgG levels per se could be used as a biomarker for CHB risk. We, however found no significant difference between the groups, with a mean total IgG level of 9.0 g/L (SD ±3.4) in the CHB_never_ group, and 10.5 g/L (SD ±4.2) in the CHB_ever_ group (Figure 2D). Nonetheless, the total IgG levels correlated significantly with levels of all three autoantibodies, most prominently with anti‐Ro60 (*r* = 0.65, *p* < 0.0001), followed by anti‐Ro52 (*r* = 0.55, *p* < 0.0001) and anti‐La (*r* = 0.46, *p* < 0.0001) (Figure 2E).

### 3.3 Autoantibody Levels Across Sequential Pregnancies

Following a pregnancy complicated by CHB, a subsequent pregnancy may or may not be complicated by CHB. To understand if the levels of autoantibodies differ systematically in succeeding pregnancies when CHB did not develop, we evaluated whether autoantibody levels changed in subsequent pregnancies following a CHB‐affected pregnancy. For three participants with available samples both from a CHB‐affected pregnancy before treatment with fetal steroids and a subsequent non‐CHB pregnancy, autoantibody levels were largely similar in the unaffected pregnancy (Figure 3A).

**FIGURE 3:**
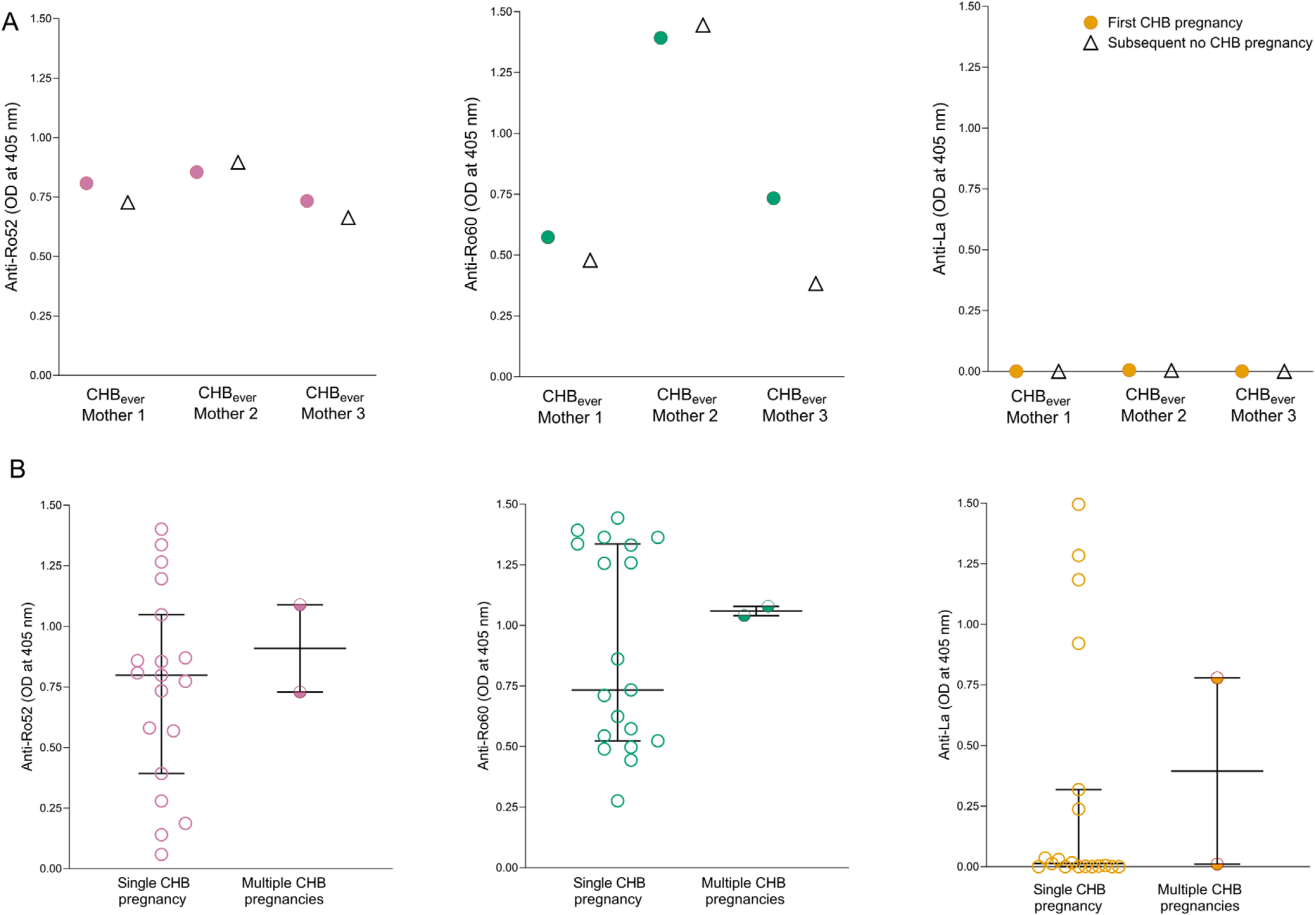
Autoantibody levels in CHB pregnancies and subsequent affected or unaffected pregnancies. (A) Autoantibody levels (optical density, OD) for anti‐Ro52, anti‐Ro60, and anti‐La during a pregnancy complicated by CHB and a subsequent pregnancy unaffected by CHB. Samples obtained closest to GW 18–24 were measured by ELISA at 1:10,000 (anti‐Ro60) or 1:2000 (anti‐Ro52 and anti‐La) dilution. Individuals had not been treated with fluorinated steroids when the sample was obtained. (B) Autoantibody levels (optical density, OD) for anti‐Ro52, anti‐Ro60, and anti‐La in women with one CHB pregnancy only and with two CHB pregnancies. No individual had been treated with steroids on fetal indication when the sample was obtained. Samples obtained closest to GW 18–24 were measured by ELISA at 1:10,000 (anti‐Ro60) or 1:2000 (anti‐Ro52 and anti‐La) dilution.

We hypothesized that recurring CHB pregnancies could relate to higher autoantibody levels and therefore compared the autoantibody levels in women with only one CHB‐affected pregnancy to those with multiple CHB pregnancies (no samples with fetal steroids). While the number of pregnancy samples from women with more than one CHB pregnancy was limited, the distribution of autoantibody levels did not reveal any trend for higher autoantibody levels in women with multiple CHB pregnancies (Figure 3B).

### 3.4 Intra‐Pregnancy Variation of Autoantibody Levels in CHB_never_ and CHB_ever_ Mothers

To understand how the autoantibody levels varied during the course of pregnancy, we next analysed pregnancies sampled longitudinally. The relative pattern of levels between autoantibodies stayed stable during and between pregnancies in both CHB_never_ and CHB_ever_ mothers (Figure 4A,B). In both groups, the pattern of autoantibody specificities remained stable throughout gestation, with a gradual decline in autoantibody levels toward delivery (Figure 4A,B), consistent with the known decrease in IgG levels during pregnancy [16]. Treatment with fetal steroids also coincided with autoantibody level decrease in several cases. Notably, autoantibody levels in the non‐pregnant state were usually similar to levels in early pregnancy.

**FIGURE 4:**
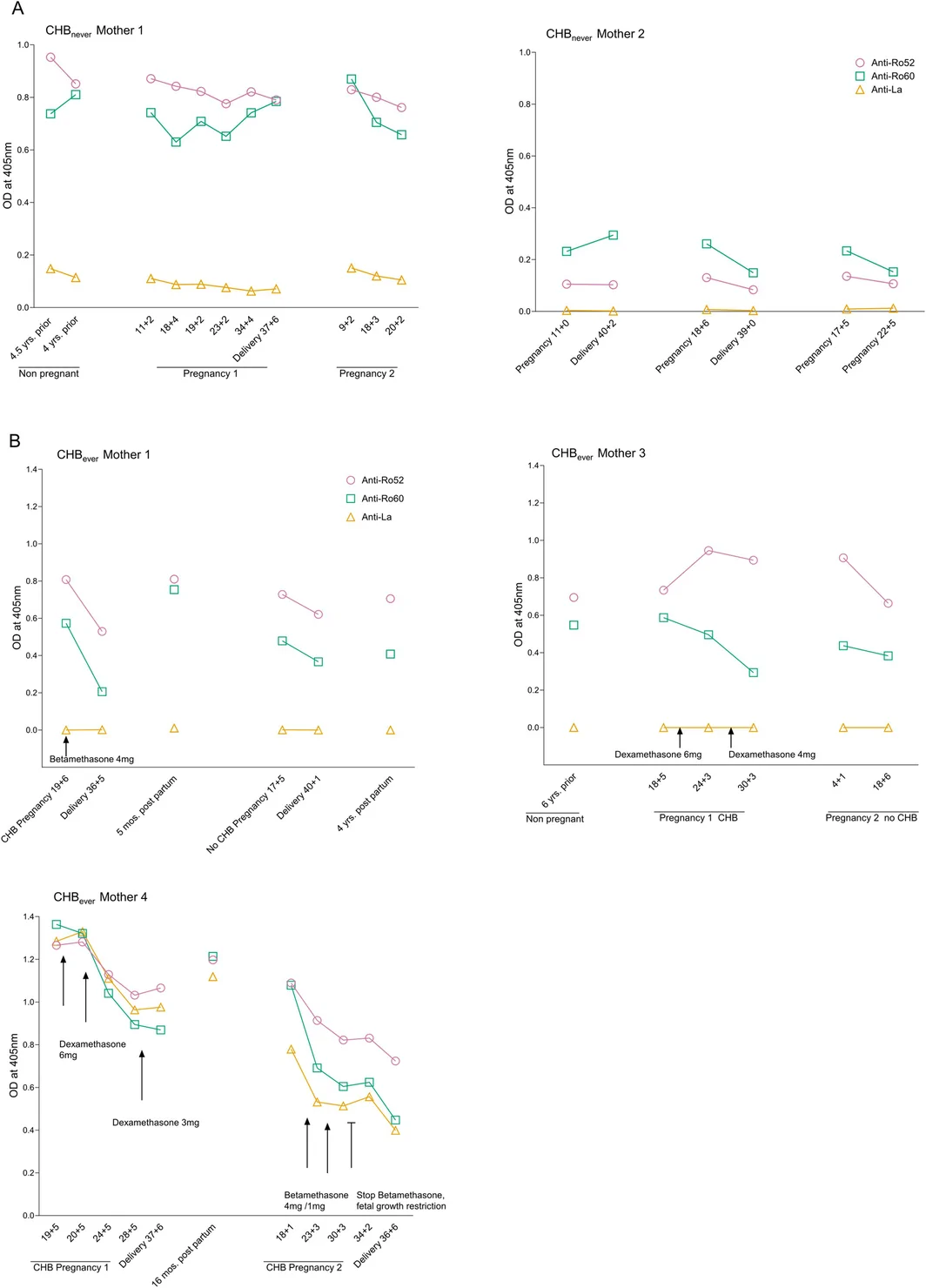
Longitudinal autoantibody levels during successive pregnancies. (A) Anti‐Ro52, anti‐Ro60, and anti‐La levels across multiple time points during several pregnancies in two women from the CHB_never_ group. Samples were measured by ELISA at 1:10,000 (anti‐Ro60) or 1:2000 (anti‐Ro52 and anti‐La) dilution. (B) Autoantibody levels over time during multiple pregnancies in three women from the CHB_ever_ group. Treatment with fluorinated steroids is indicated by arrows. Samples were measured by ELISA at 1:10,000 (anti‐Ro60) or 1:2000 (anti‐Ro52 and anti‐La) dilution.

### 3.5 Anti‐Ro60 Levels Have the Highest Discriminatory Performance in Predicting CHB Risk

Anti‐Ro60 and anti‐Ro52 levels were significantly higher in women who had experienced a CHB‐affected pregnancy than in non‐affected women, while anti‐La antibodies did not differ between groups (Figure 2B). Risk stratification based on deciles of autoantibody levels demonstrated marked case enrichment in the highest risk decile compared with the lowest decile, predominantly for anti‐Ro60 antibodies, but also for anti‐Ro52 antibodies (Figure 5A). For anti‐Ro60 antibodies, the relative risk (RR) was 16.3 (95% CI 1.04–255.0, *p* < 0.001) for the top versus the bottom decile, and for anti‐Ro52 the RR was 11.9 (95% CI 0.73–193.4, ns) for the top versus the bottom decile. We next analysed which combination of specificities and the level at which the autoantibodies had the most effect on CHB risk using a supervised machine‐learning framework based on logistic and LASSO regression (Figure 5B). Increasing composite antibody scores were associated with higher odds of case status overall, with the largest OR per 1SD increase being 3.2 (95% CI 1.8–6.3) when using levels of all three autoantibodies and total IgG. However, the magnitude of the ORs increased only marginally as additional biomarkers were incorporated into the models, as models using anti‐Ro60 levels alone had an OR per 1SD increase of 2.8 (95% CI 1.6–5.3). Thus, among all evaluated models, anti‐Ro60 levels alone showed the highest cross‐validated discriminatory performance (Figure 5B), and for a sensitivity of 0.95 generated a specificity of 0.54 with an AUC of 0.76 in ROC analysis (Figure 5C).

**FIGURE 5:**
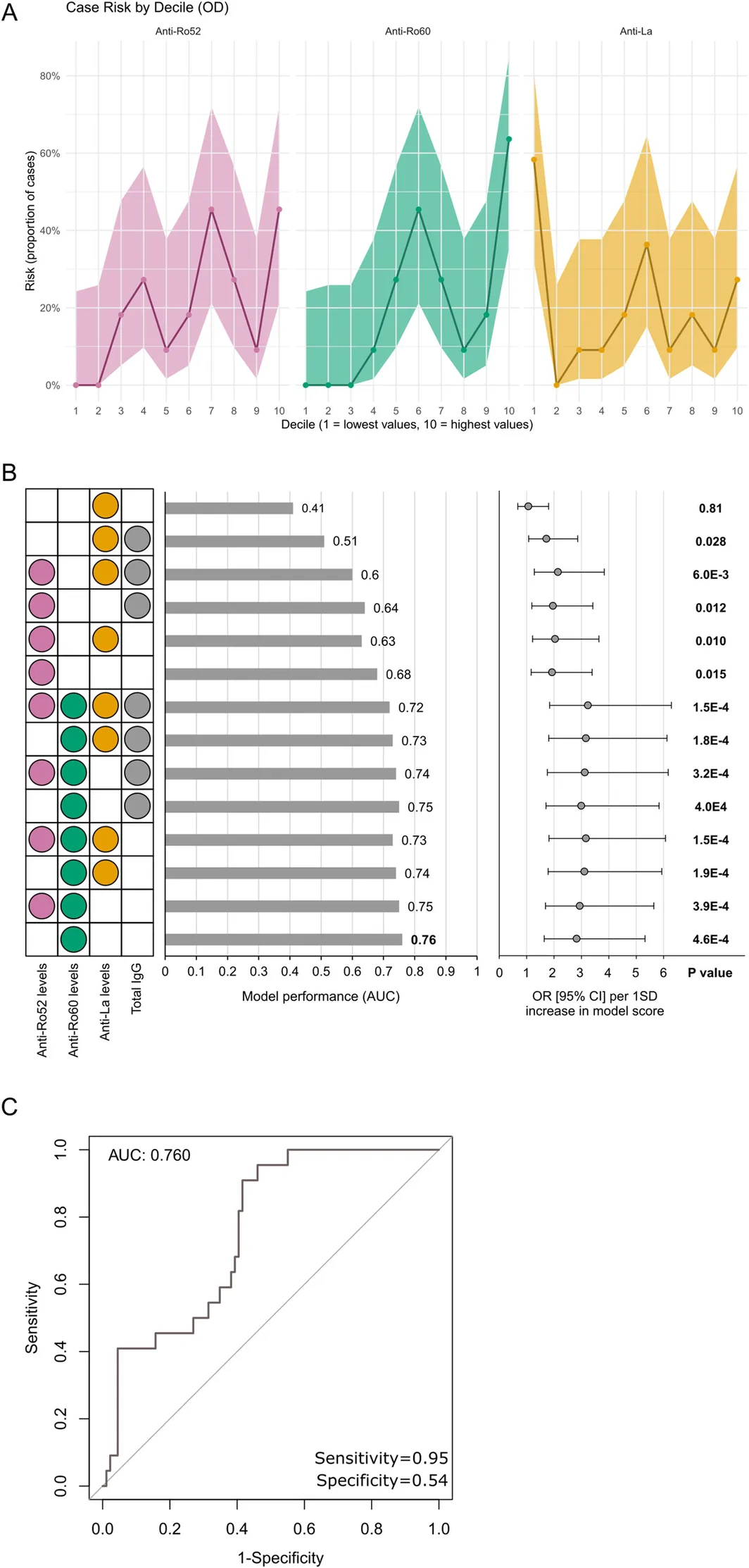
Risk of CHB based on autoantibody levels in samples from 89 CHB_never_ and 22 CHB_ever_ pregnancies. (A) Case risk by deciles of respective autoantibody level defined by OD value. For anti‐Ro60 antibody levels, the relative risk (RR) was 16.3 (95% CI 1.04–255.0, *p* = 0.001) for the top versus the bottom decile (10th versus 1st decile), and for anti‐Ro52 antibody levels the RR was 11.9 (95% CI 0.73–193.4, ns) for the top versus the bottom decile (10th vs. 1st decile). (B) AUC values from the logistic and LASSO models (left) and OR with 95% CI of risk status per 1 standard deviation (SD) increase in model score (right) for all antibody level combinations. (C) Receiver‐operating characteristic (ROC) curve of the logistic model with the highest predictive value.

## 4 Discussion

In this study, we investigated the relationship between CHB and maternal levels of anti‐Ro52, anti‐Ro60, and anti‐La autoantibodies to assess their potential as biomarkers for fetal AV block risk in autoantibody‐positive women.

In clinical settings, anti‐Ro/La autoantibody testing typically focuses on determining the presence or absence of reactivity using low sample dilutions, with limited assessment of antibody titres beyond a positive threshold [17]. However, high‐titre autoantibodies—potentially relevant for fetal outcomes—are not adequately captured by this approach. Leveraging samples from a national cross‐sectional cohort and a longitudinally followed local cohort, we were able to evaluate antibody levels from a large number of anti‐Ro/La autoantibody‐positive women giving, or not giving, birth to children with CHB, with a subset of pregnancies followed short‐ or long‐term at our centre over the past 20 years.

When comparing antibody positivity or negativity established by clinical routine testing in our study, we found a higher frequency of anti‐Ro60 antibodies in autoantibody‐positive women who had experienced a CHB pregnancy compared to those who had not. While statistically significant, the difference was small. As anti‐Ro52 positivity was an inclusion criterion in our prospectively followed cohort for the main part of the period, almost all women were positive for this antibody, which limited our ability to assess its relationship to CHB. Autoantibodies can occur at very high titres, but routine testing commonly does not quantify antibody levels over a certain cutoff [17]. Previous studies suggest that antibody titres, not just presence, may better reflect CHB risk [12, 13, 14, 18], and we therefore titrated samples to define dilutions for each autoantibody specificity that discriminated antibody levels for high‐titre samples. We found that a dilution of 1:10,000 was needed to accurately define anti‐Ro60 antibodies, reflecting the extremely high levels of these autoantibodies.

Our results from high‐dilution ELISA testing to assess whether autoantibody levels were associated with CHB demonstrate that high levels of anti‐Ro60 autoantibodies are strong and independent predictors of CHB risk in anti‐Ro/SSA positive pregnancies, and also serve to define pregnancies without risk. In contrast, anti‐Ro52 levels, while elevated in affected individuals, did not contribute significantly to predictive models. This may at least in part relate to the fact that anti‐Ro52 positivity was an inclusion criterium for the surveillance program building the prospective cohort for most of the period. Level of anti‐La did not differ between groups and did not further inform on risk. Our data are in agreement with observations from Buyon et al. [12], Jaeggi et al. [13], and Kaizer et al. [14], who all found that the risk of CHB is associated with antibody levels at the higher end of the spectrum, and that there may be a group with low Ro52 and low anti‐Ro60 antibodies without risk even if they test positive for these autoantibodies. Our findings in the present study add to the nuances in interpretation of antibody profiles in maternal screening, and provide a potential framework for establishing quantitative thresholds that could inform clinical risk counselling.

By employing traditional and machine learning regression models and evaluating their predictive value, we provide evidence that antibody levels, rather than simple seropositivity, offer enhanced resolution for risk stratification. However, while ROC‐based analysis showed excellent sensitivity (95.4%) at a threshold of 1 in identifying women who had experienced CHB pregnancies, the specificity was more modest, with approximately half of the CHB_never_ group classified as high‐risk. This underlines an important clinical reality: high maternal autoantibody levels are a necessary but insufficient prerequisite for CHB development, as illustrated by the low recurrence rate of only 12%–16% despite persistent maternal autoantibodies [5, 6, 10]. These observations support the notion that additional fetal or maternal factors modulate disease expression in foetuses exposed to anti‐Ro/La antibodies. In this context, reports have identified both MHC and non‐MHC genes of which fetal polymorphisms contribute to the risk of CHB [19, 20, 21]. Although less explored, environmental and lifestyle factors could potentially also contribute [22].

The main limitation of our study is the number of samples taken during pregnancies affected by CHB. However, the condition is rare, and our collective dataset represents one of the largest and most comprehensively followed maternal CHB cohorts. We also used novel approaches in methodology, including high sample dilution in testing and advanced modelling for analysis. Another limitation worth noting is the inclusion criterium of anti‐Ro52 positivity for the main part of the surveillance program and prospectively collected samples, which restricts the discrimination power of those antibodies in the current study and may explain why they did not contribute more to the risk in prediction modelling.

Together, our data support a model where high levels of anti‐Ro60 confer significant CHB risk, while the levels of anti‐Ro52 and anti‐La did not further inform risk. The predictive models developed here offer a quantitative and biologically grounded approach to risk stratification and have potential implications for clinical decision‐making in maternal‐fetal medicine. The observations further suggest that autoantibody analysis at high sample dilution should be considered for implementation in routine laboratory testing of women of reproductive age to better define the group with risk of CHB.

## Author Contributions

The study was designed by Lisa Rudolph, Gudny Ella Thorlacius, Sven‐Erik Sonesson and Marie Wahren‐Herlenius. Experimental work was performed by Lisa Rudolph, Elina Richardsdotter Möller and Vijole Ottosson, and statistical analyses were performed by Lisa Rudolph and Gudny Ella Thorlacius with input from Rui Da Silva Rodrigues, Gregoire Martin de Fremont, Sven‐Erik Sonesson and Marie Wahren‐Herlenius. Linda Lagnefeld, Iva Gunnarsson, Elisabet Svenungsson, Felicia Nordenstam, Gunnar Bergman and Sven‐Erik Sonesson recruited and included the patients in the study. Lisa Rudolph wrote the first draft of the manuscript with input from the coauthors and under the supervision of Sven‐Erik Sonesson, Gudny Ella Thorlacius and Marie Wahren‐Herlenius, and all authors contributed to editing the final version.

## Funding

This work was supported by Vetenskapsrådet, 2021‐02503. Hjärt‐Lungfonden. Stockholms Läns. Landsting. Norges Forskningsråd. Reumatikerförbundet. Knut och Alice Wallenbergs Stiftelse. Stiftelsen Konung Gustaf V:s 80‐årsfond. FOREUM Foundation for Research in Rheumatology.

Fonds de dotation Villa M. Janssen Pharmaceutica NV.

## Ethics Statement

The study was approved by the Regional Ethics Committee Stockholm, and participants gave written informed consent.

## Conflicts of Interest

Marie Wahren‐Herlenius obtained funding from Janssen Pharmaceutica NV. Gudny Ella Thorlacius is currently employed by Amgen deCODE Genetics, Reykjavik, Iceland.

## Data Availability

The data that support the findings of this study are available on request from the corresponding author. The data are not publicly available due to privacy or ethical restrictions.
